# Whole grain food diet slightly reduces cardiovascular risks in obese/overweight adults: a systematic review and meta-analysis

**DOI:** 10.1186/s12872-020-01337-z

**Published:** 2020-02-18

**Authors:** Weihao Wang, Jianan Li, Xiaoxi Chen, Miao Yu, Qi Pan, Lixin Guo

**Affiliations:** 1 Department of Endocrinology, Beijing Hospital, National Center of Gerontology, Institute of Geriatric Medicine, Chinese Academy of Medical Sciences, Beijing, People’s Republic of China; 2 Department of Cardiology, Fuwai Hospital, National Center for Cardiovascular Diseases, Chinese Academy of Medical Sciences and Peking Union Medical College, Beijing, China

**Keywords:** Whole grain food, Cardiovascular risk factors, Overweight adults, Systematic review, Body weight

## Abstract

**Background:**

The effects of whole grain diet on cardiovascular risks in obese and overweight adults is not well established. Our goal was to conduct a systematic review and meta-analysis on the effect of whole grain diet on cardiovascular risks in obese/overweight adults.

**Methods:**

PubMed, Embase and Cochrane were systematically scanned for randomized controlled trials (RCTs), and studies were selected based on certain inclusion and exclusion criteria. The primary outcome was the effectiveness of whole grain food consumption in reducing body weight. The secondary outcomes were the effect of whole grain food consumption on cardiovascular disease (CVD) risk factors including plasma low-density lipoprotein cholesterol (LDL-C), insulin resistance index, blood pressure, body mass index (BMI), C-reactive protein (CRP), and waist circumference in obese/overweight adults.

**Results:**

Our results showed that whole grain consumption was associated with lower body weight (mean difference (MD) = − 0.5, 95% confidence intervals (CI) [− 0.74, 0.25], I^2^ = 35%, *P* < 0.0001) and lower CRP (MD = -0.36, 95% CI [− 0.54, − 0.18], I^2^ = 69%, P < 0.0001), compared with the control group. However, there were no significant differences in LDL-C (MD = -0.08, 95% CI [− 0.16, 0.00], I^2^ = 27%, *P* = 0.05), waist circumference (MD = -0.12, 95% CI [− 0.92, 0.68], I^2^ = 44%, *P* = 0.76), systolic blood pressure (MD = -0.11, 95% CI [− 1.55, 1.33], I^2^ = 3%, *P* = 0.88), diastolic blood pressure (MD = -0.44, 95% CI [− 1.44, 0.57], I^2^ = 15%, *P* = 0.39), and fasting glucose (MD = -0.05, 95% CI [− 0.12, 0.01], I^2^ = 31%, *P* = 0.11) between the two groups.

**Conclusion:**

This study suggests that whole grain food consumption can slightly reduce body weight and CRP in obese/overweight population.

## Background

Cardiovascular diseases (CVD) remain the leading cause of morbidity and mortality in the world, including in China, wherein it accounts for around one third of all deaths [1]. Overweight and obesity are global health problems, their scope and severity are growing [2], and there is a high demand for a global health care system to overcome their outcomes. Overweight and obesity management and diets are core approaches in all clinical practice guidelines for reducing the risk of CVD. Besides, nutritional plans to achieve optimal body weight are important for preventing obesity-related diseases.

Observational studies have shown that increased consumption of whole grain foods was associated with lower incidence of metabolic syndrome [3] and lower mortality rates from CVD [4–7]. In addition, studies have shown that whole grain foods exert beneficial effects on glucose metabolism, obesity, blood pressure, body lipids and inflammatory markers [8–11]. Indeed, whole grain foods are recommended for the prevention of CVD due to their cardioprotective content including dietary fibers, trace minerals, phytoestrogens and antioxidants [12, 13]. Besides, whole grain foods are thought to ameliorate body weight due to their lower energy density and satiety, compared with refined grain foods [5, 14–19].

Several observational studies demonstrated that high whole grain food consumption is associated with lower BMI [20, 21] and lower long-term weight gain [22, 23]. In contrast, a recent meta-analysis showed that whole grain intake may have a slight beneficial effect on body fat mass, with no significant effect on body weight [9]. Similarly, a recent systematic review pointed to inconsistent evidence between intervention studies on the effect of whole grain food consumption on weight loss, independent of caloric restriction [24]. Hence, the overall conclusion from current studies suggesting that whole grain food consumption can reduce body weight is relatively inconsistent and warrant further investigation. Therefore, we aimed in this study to evaluate the impact of whole grain food consumption on cardiovascular risk factors in overweight and obese patients through the meta-analysis of related randomized controlled trials.

## Methods

### Data sources

The meta-analysis was performed according to the Cochrane Handbook for Systematic Reviews of Interventions and Preferred Reporting Items for Systematic Reviews and Meta-Analyses (PRISMA). Briefly, PubMed, Embase and Cochrane Central Register of Controlled Trials databases were scanned for RCTs without time and race restriction. PubMed was queried using the following advanced search query: ((((((((obesity) [Abstract] OR obese) [Abstract] OR overweight) [Abstract] OR fat)) [Abstract] OR metabolic syndrome [Abstract])) AND (((randomized controlled trial) [Abstract] OR placebo) [Abstract] OR randomized [Abstract])) AND ((((Grain, Whole) [Abstract] OR Grains, Whole) [Abstract] OR Whole Grain) [Abstract] OR Grain Cereal, Whole [Abstract]).

### Inclusion and exclusion criteria

Studies were included if they satisfied the following inclusion criteria: (1) intervention time lasted more than 2 weeks; (2) randomized controlled trial; (3) assessing cardiovascular outcomes in obese/overweight adults (BMI ≥ 24 kg/m^2^); (4) at least one of the following secondary outcomes were measured: weight, blood pressure, BMI, waist circumference and cholesterol. A study was excluded if: it was not an RCT, a review or a meta-analysis, it was a case report, the study time lasted less than 2 weeks, and the reported information were inadequate.

The following data were extracted from each record by two independent researchers (WWH and LJN): study characteristics (author, publication year, duration of intervention time, sample size), baseline characteristics of patients (age, body weight, waist circumference, BMI, systolic blood pressure (SBP), LDL-C), and cardiovascular risk outcomes including body weight (primary outcome) and other secondary outcomes such as plasma low-density lipoprotein-cholesterol level, insulin resistance index, blood pressure (BP), BMI, CRP, waist circumference .

### Assessment of bias

Assessment of bias was analyzed using the Cochrane Collaboration’s tool. The following factors were assessed: random sequence generation (selection bias), allocation concealment (selection bias), blinding of participants and personnel (performance bias), blinding of outcome assessment (detection bias), incomplete outcome data (attrition bias), selective reporting (reporting bias), and other bias. Two independent investigators (CXX and YM) evaluated the bias in the included articles.

### Statistical analysis

Statistical analysis was performed using the Review Manager (Revman). The Odds ratios (OR) and 95% confidence intervals (CI) were calculated for dichotomous outcomes, while the mean difference (MD) and 95% CI were calculated for continuous outcomes. A *p*-value less than 0.05 was used as a cutoff for statistical significance. The random effect model was used to generate forest plots. The I^2^ test was used to assess data heterogeneity, where an I^2^ more than 50% indicated highly heterogeneous results. Subgroup analysis was conducted on positive results to investigate the source of heterogeneity.

## Results

### The characteristics of included studies

**Fig. 1:**
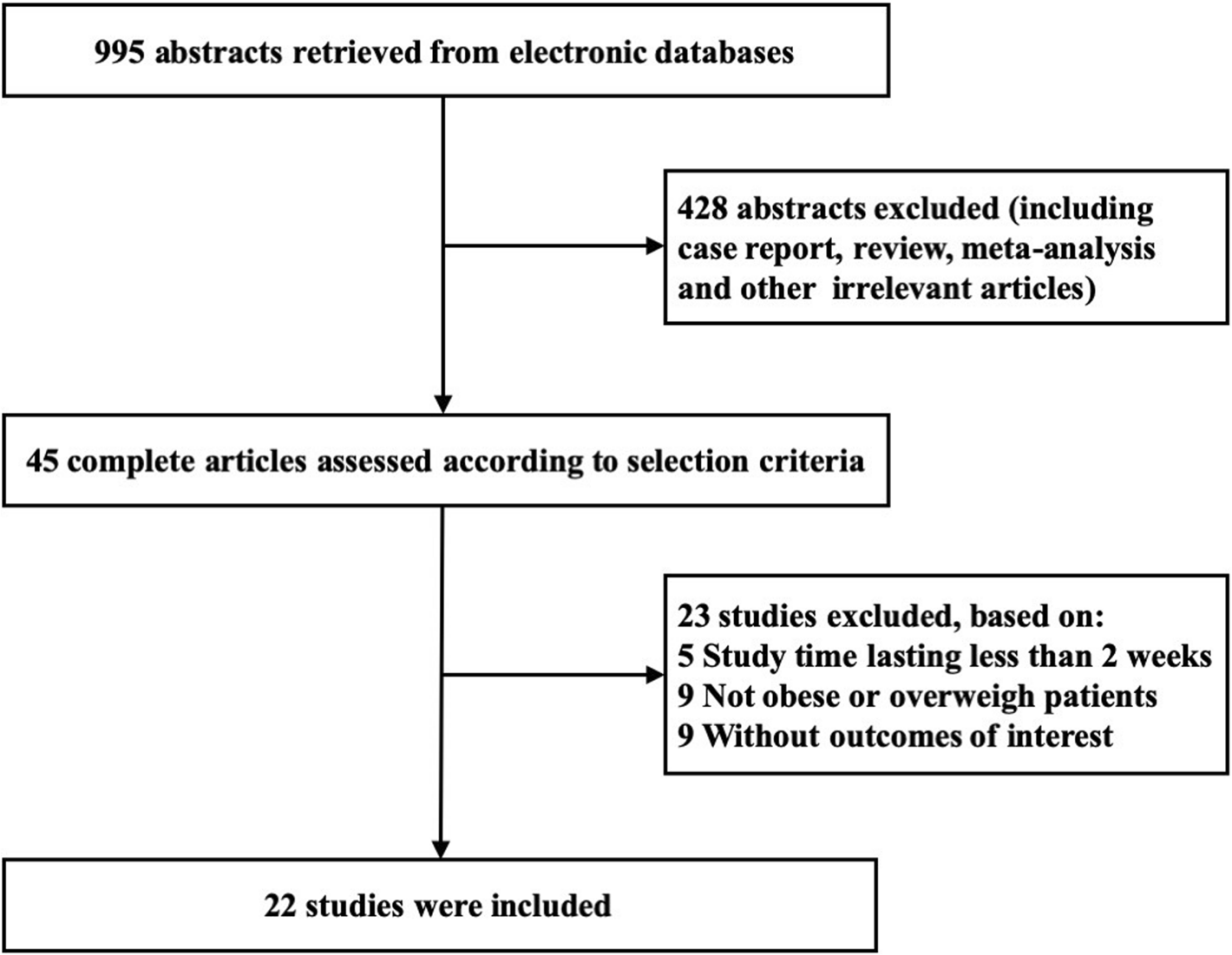
Flow diagram of study inclusion and exclusion criteria

A total of 995 studies were identified by the two investigators, including 518 articles from PubMed, 145 from Embase, and 332 from Cochrane database. From these studies, 45 relevant studies were selected for detailed evaluation, of which 22 randomized clinical trials were finally included for the meta-analysis. The screening process for eligible studies is shown in Fig. 1. The characteristics of studies and populations included in the meta-analysis are present in Table 1. The relevant information for each RCT were collected, including authors, year, intervention and trial duration, as well as subjects’ age, number, mean body weight, mean waist circumference, mean BMI, mean SBP and mean LDL-C.

**Table 1:** Main characteristics of included studies. RCT, randomized controlled trial; BMI, body mass index; SBP, systolic blood pressure; LDL, low density lipoprotein; NA, not available

Author, Year	Study design	Intervention	Duration of trial (week)	Number	Age	Mean Body weight (kg)	Mean Waist circumference (cm)	Mean BMI (kg, m^2^)	Mean SBP (mmHg)	Mean LDL (mmol, L)
Katcher, 2008 [25]	RCT	Whole grain	12	50	NA	103.1 vs 106.2	82 vs 83.2	NA	123 vs 130	2.83 vs 2.93
K. Rave, 2007 [26]	RCT	Whole grain	4	NA	NA	97.5 vs 98.8	NA	33.2 vs 33.7	131 vs 134	3.8 vs 3.8
HARRIS JACKSON, 2014 [27]	RCT	Whole grain	12	25 VS 25	45.8 vs 46.4	99.5 vs 99.7	NA	33.5 vs 32.9	NA	NA
Maria Lankinen, 2014 [28]	RCT	Whole grain	12	34 Vs 35	NA	NA	106.3 vs 105.8	31.4 vs 31	135 vs 139	3.2 vs 3.4
Roager HM, 2017 [29]	RCT	Whole grain	8	50 Vs 50	NA	85.4 vs 86.1	100.1 vs 100.4	NA	126.2 vs 124.2	3.2 vs 3.2
Kirwan, 2016 [30]	RCT	Whole grain	8	33 Vs 33	NA	93.2 vs 93.7	96.4 vs 95.5	32.9 vs 33.1	NA	2.76 vs 2.76
I.A.Brownlee, 2010 [31]	RCT	Whole grain	16	33 VS 33	45.9 vs 45.6	86.7 vs 86.7	NA	30 vs 30	125.5 vs 127.3	3.2 vs 3.2
Steven K. Malin, 2018 [32]	RCT	Whole grain	8	14 VS 14	37.9 vs 37.9	97.9 vs 97.9	NA	33.8 vs 33.9	NA	NA
SCHUTTE, 2018 [33]	RCT	Whole grain	12	25 vs 25	61 vs 61	84.6 vs 86.2	102.2 vs 103.4	27.6 vs 28	NA	NA
Bernard J. Venn, 2010 [34]	RCT	Whole grain	72	53 vs 55	42 vs 42	99 vs 95	NA	36.1 vs 34.7	NA	NA
P. Hajihashemi, 2014 [35]	RCT	Whole grain	6	44 vs 44	11.2 vs 11.2	51.26 vs 51.26	80.69 vs 80.69	23.57 vs 23.57	NA	NA
K. Nelson, 2016 [36]	RCT	Whole grain	4	10 vs 10	NA	NA	NA	30.77 vs 31	133.3 vs 132.1	3.27 vs 3.12
Paula Tighe, 2013 [37]	RCT	Whole grain	12	73 vs 63	51.6 vs 51.8	NA	85.7 vs 90.9	28 vs 28	125.9 vs 131.2	3.45 vs 3.66
Mette Kristensen, 2017 [38]	RCT	Whole grain	12	30 vs 30	NA	NA	NA	28.5 vs 28.4	130 vs 130	3.7 vs 3.7
S. FATAHI, 2018 [39]	RCT	Whole grain, fruits and vegetables, both	10	25 vs 25 vs 25	36.7 vs 34.6 vs 39.9	NA	NA	32.1 vs 32.3 vs 32.7	NA	NA
Xue Li, 2016 [40]	RCT	Whole grain	4	60 vs 79 vs 80 vs 79	59 vs 59.73 vs 59.72 vs 59.44	NA	NA	NA	143.7 vs 147.2 vs 144.9 vs 147.1	NA
Kevin C. Maki, 2010 [41]	RCT	Whole grain	12	77 vs 67	50.1 vs 47.5	88.7 vs 87.6	104.5 vs 105.2	32 vs 32.2	NA	4 vs 3.99
V. D. F. de Mello, 2011 [42]	RCT	Whole grain, Healthy diet	12	34 vs 36 vs 34	58 vs 59 vs 59	89.2 vs 89.8 vs 89.5	106.3 vs 105.7 vs 105.7	31.4 vs 31.1 vs 30.9	135 vs 138 vs 139	3.2 vs 3.1 vs 3.4
A. Stefoskaneedham, 2017 [43]	RCT	Whole grain	12	30 vs 30	48.1 vs 48.6	87.1 vs 86.1	102.5 vs 105.3	31.2 vs 31.6	122.3 vs 126.2	3.2 vs 3.5
Kristensen, 2012 [44]	RCT	Whole grain	12	38 vs 34	NA	81.3 vs 83.5	97.3 vs 99	30 vs 30.4	133 vs 138	3.75 vs 3.75
J. Tovar, 2014 [45]	RCT	Whole grain	4	26 vs 26	NA	NA	NA	NA	NA	NA
Mette Kristensen, 2017 [38]	RCT	Whole grain	12	81 vs 88	36.2 vs 35.3	80.2 vs 81.5	NA	30.2 vs 30.1	109.8 vs 111.2	2.9 vs 2.72

### Cardiovascular outcomes

**Fig. 2:**
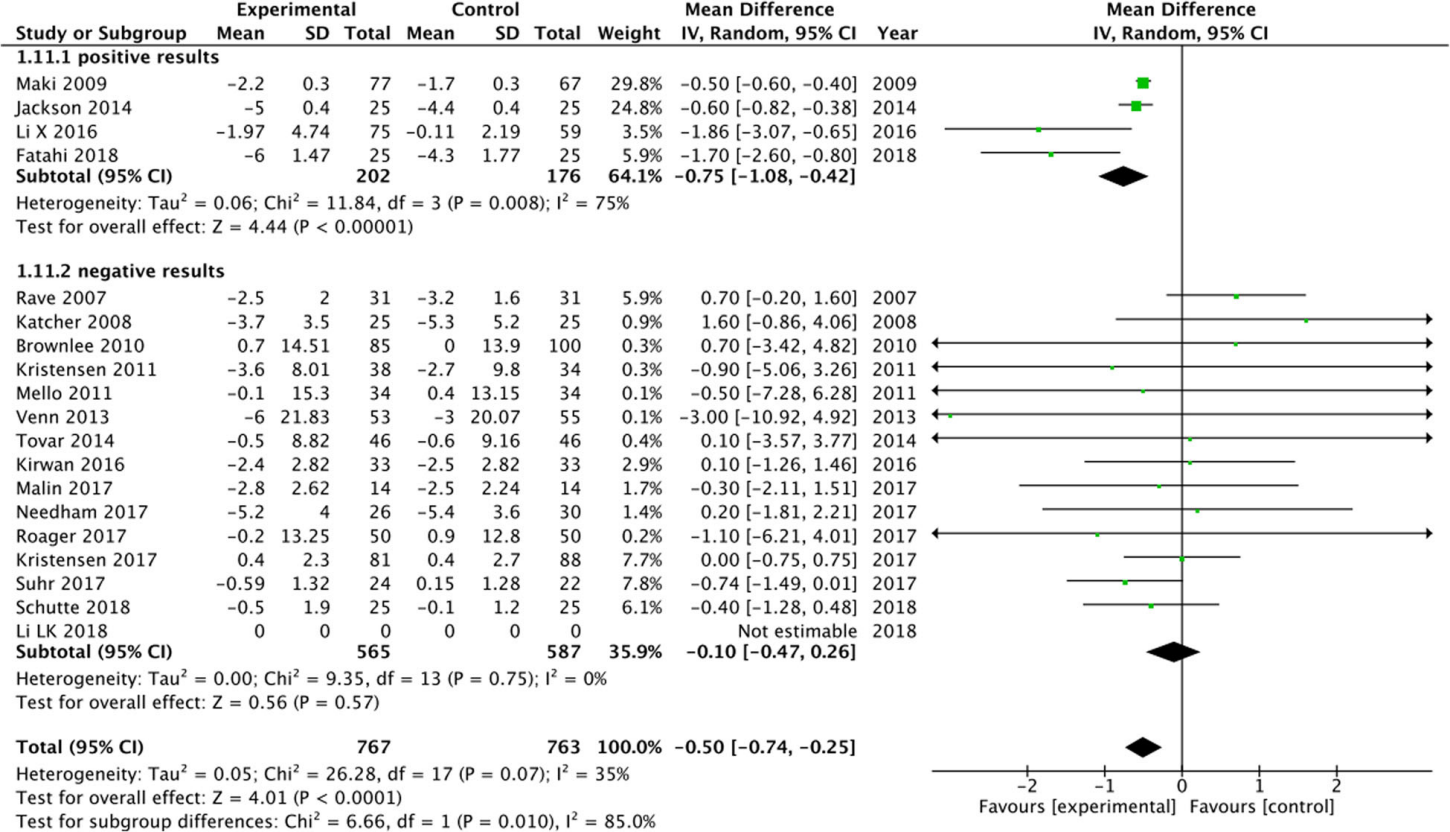
Forest plot for meta-analysis comparing whole grain with placebo in weight. CI: confidence interval; SD: standard deviation; df: degrees of freedom; IV: inverse variance

**Fig. 3:**
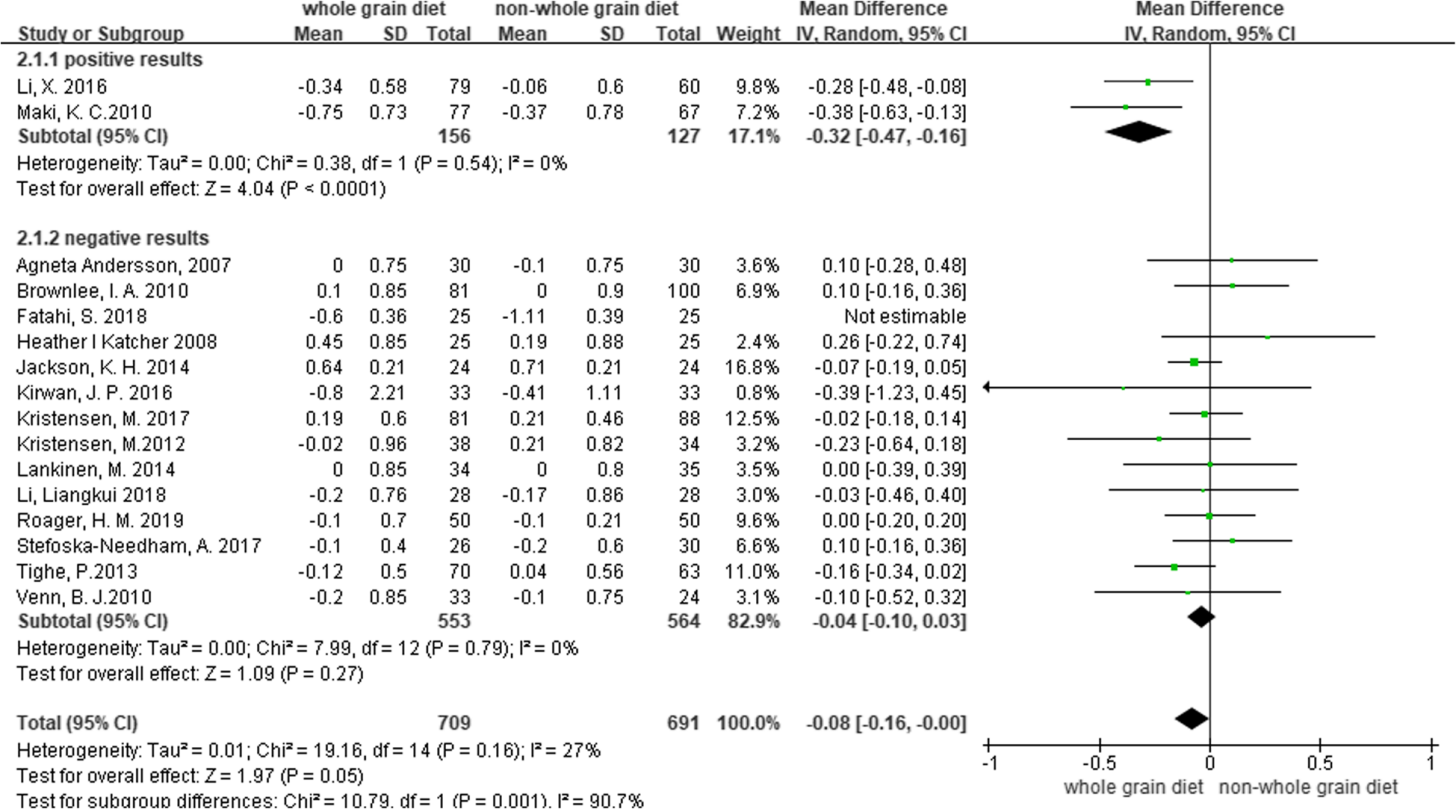
Forest plot for meta-analysis comparing whole grain with placebo in C-reactive protein (CRP). CI: confidence interval; SD: standard deviation; df: degrees of freedom; IV: inverse variance

**Fig. 4:**
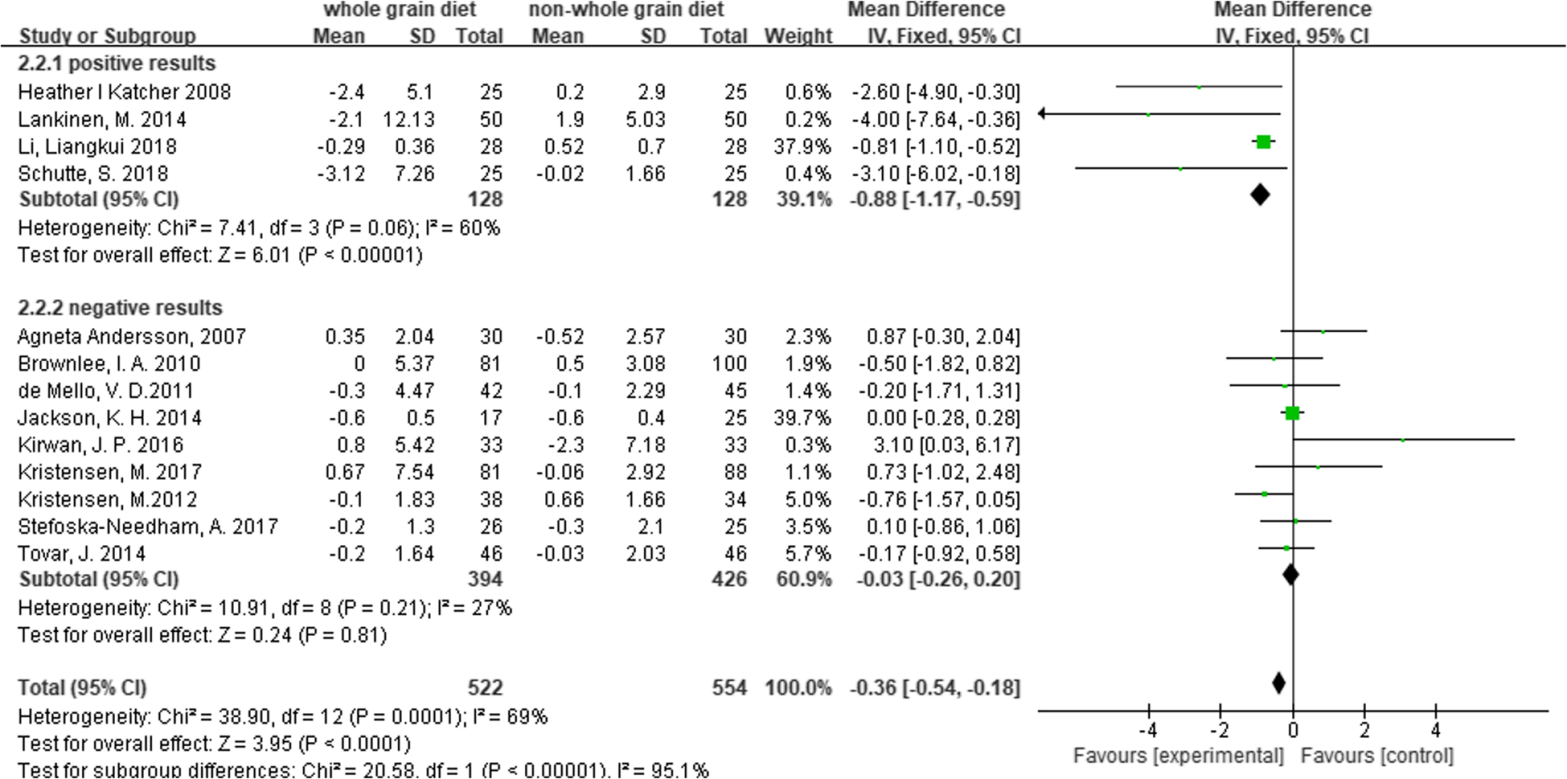
Forest plot for meta-analysis comparing whole grain with placebo in low density lipoprotein cholesterol (LDL-C). CI: confidence interval; SD: standard deviation; df: degrees of freedom; IV: inverse variance

**Fig. 5:**
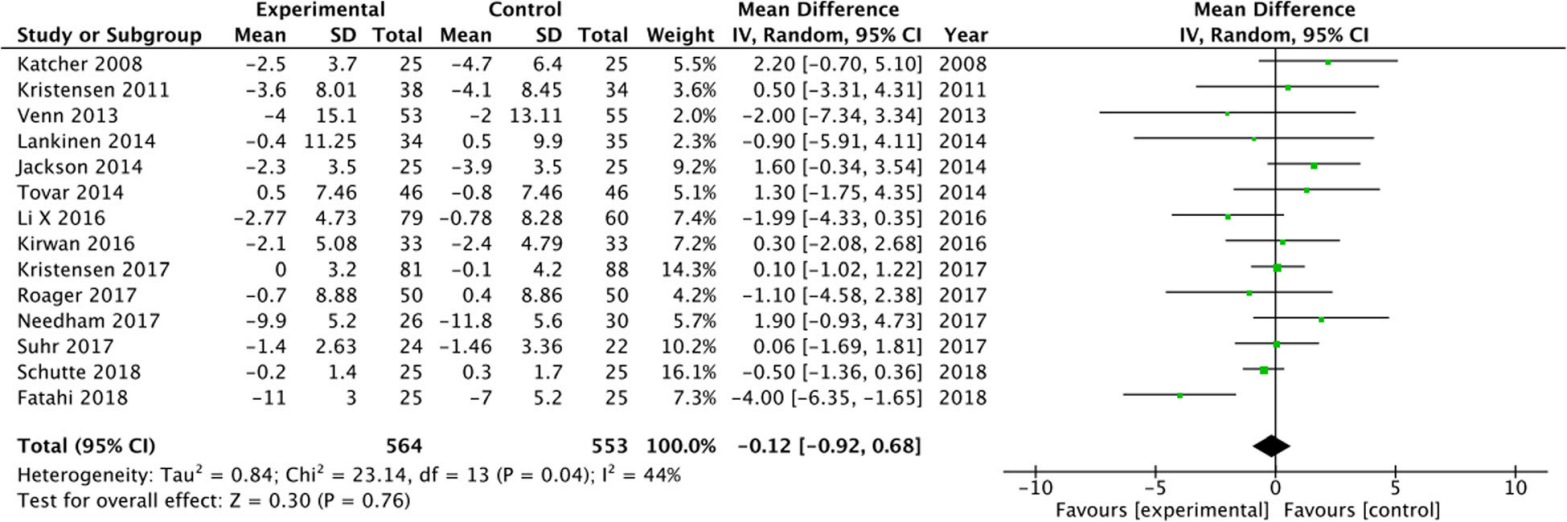
Forest plot for meta-analysis comparing whole grain with placebo in waist circumference. CI: confidence interval; SD: standard deviation; df: degrees of freedom; IV: inverse variance

**Fig. 6:**
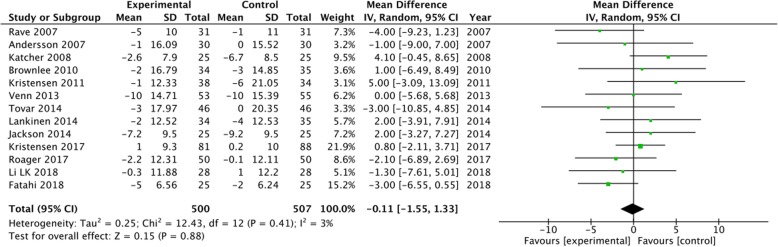
Forest plot for meta-analysis comparing whole grain with placebo in systolic blood pressure. CI: confidence interval; SD: standard deviation; df: degrees of freedom; IV: inverse variance

**Fig. 7:**
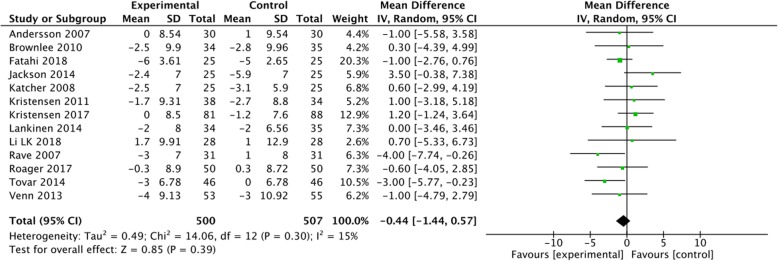
Forest plot for meta-analysis comparing whole grain with placebo in diastolic blood pressure. CI: confidence interval; SD: standard deviation; df: degrees of freedom; IV: inverse variance

**Fig. 8:**
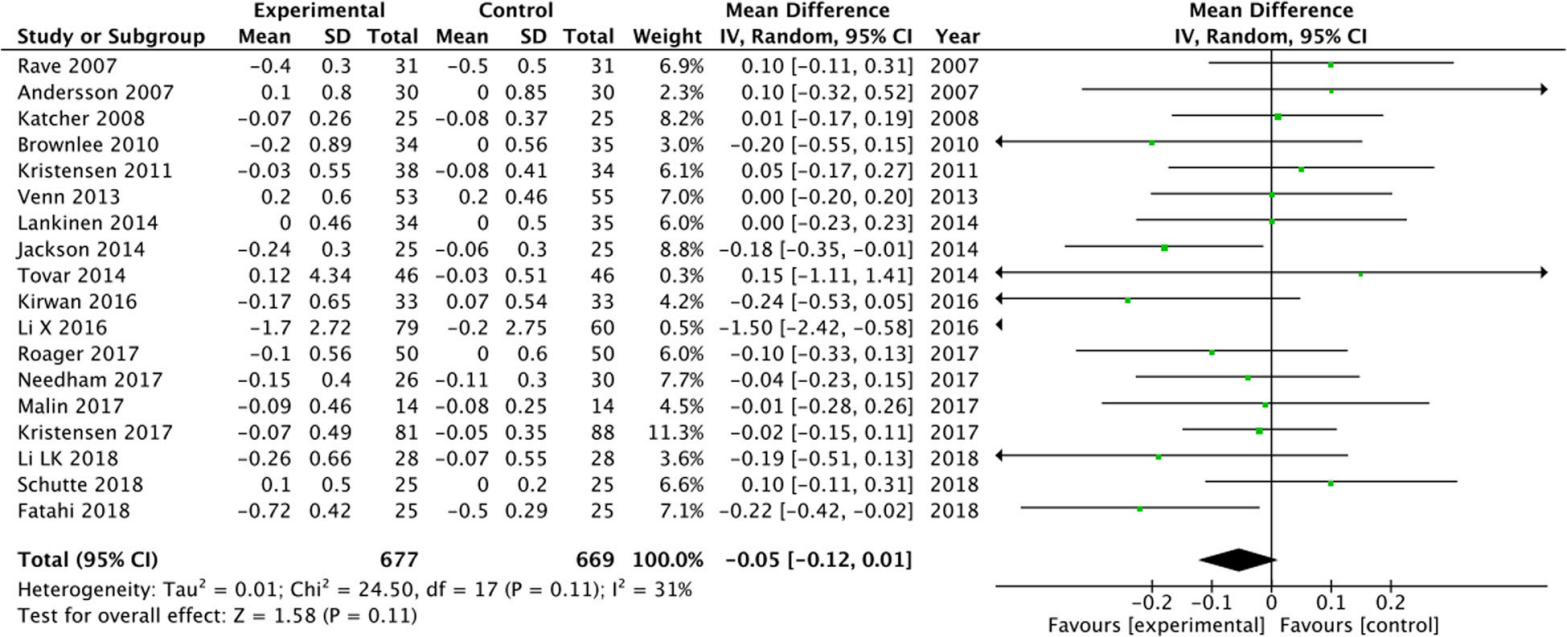
Forest plot for meta-analysis comparing whole grain with placebo in fasting glucose. CI: confidence interval; SD: standard deviation; df: degrees of freedom; IV: inverse variance

A subgroup analysis was performed for weight, LDL-C and CRP based on the positive results measured by 95% CIs. The results showed a significant decrease in body weight (MD = -0.5, 95% CI [− 0.74, − 0.25], I^2^ = 35, *P* < 0.0001) and CRP (MD = -0.36, 95% CI [− 0.54, − 0.18], I^2^ = 69%, *P* < 0.0001) in the whole grain group, compared with the control group (Figs 2, 3). However, there were no significant differences between the two groups with regard to LDL-C (MD = -0.08, 95% CI [− 0.16, 0.00], I^2^ = 27%, *P* = 0.05), waist circumference (MD = -0.12, 95% CI [− 0.92, 0.68], I^2^ = 44%, *P* = 0.76), SBP (MD = -0.11, 95% CI [− 1.55, 1.33], I^2^ = 3%, *P* = 0.88), diastolic blood pressure (DBP) (MD = -0.44, 95% CI [− 1.44, 0.57], I^2^ = 15%, *P* = 0.39), and fasting glucose levels (MD = -0.05, 95% CI [− 0.12, 0.01], I^2^ = 31%, *P* = 0.11) (Figs. 4, 5, 6, 7 and 8).

### Assessment of bias

**Fig. 9:**
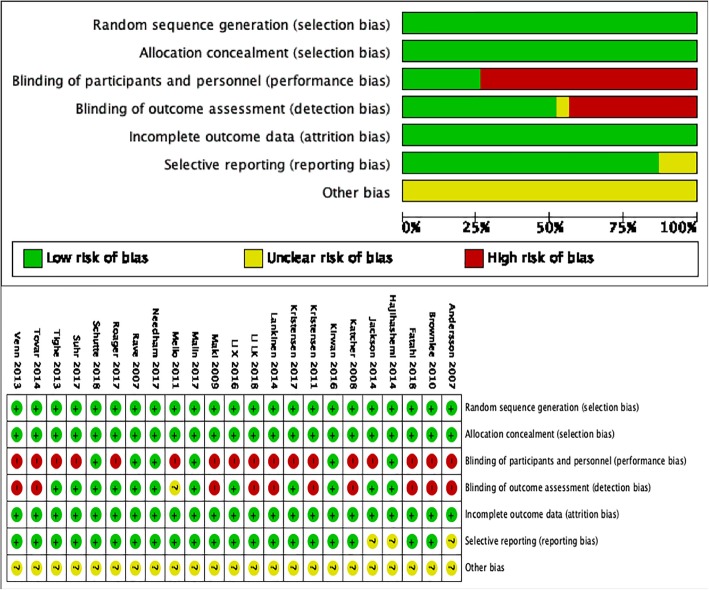
Bias Assessment of included studies. Red color: high risk of bias; Yellow color: unclear risk of bias; Green color: low risk of bias

Risk of bias in the included studies was assessed using Revman (Fig. 9). Some studies had high performance bias because they failed to implement blind intervention on subjects due to dietary intervention. However, no other significant sources of bias were observed.

## Discussion

To our knowledge, this meta-analysis is the first to evaluate the impact of whole grain diet on cardiovascular risk factors in obese/overweight adults. Our analysis suggests that whole grain diet is associated with a decrease in body weight and CRP, compared with the control group, with no significant differences between the two groups in LDL-C, waist circumference, SBP, DBP and fasting glucose levels. This meta-analysis supports previous studies recommending whole grain diet for CVD patients.

CVD remain the leading cause of mortality worldwide, and obesity is a main risk factor for the pandemic of CVD. Slavin et al. reported the protective mechanisms of whole grain diet in CVD [46], including antioxidant effects and alteration of gut environment by dietary fibers, carbohydrates and short-chain fatty acid, but also by regulating glucose metabolism and response to insulin. By revealing these mechanisms, whole grains consumption was recommended in the Australia dietary guidelines in 2003 [47]. In the 2013 edition, the guidelines highlighted that at least 4 to 6 serves per day of grains food, mainly whole grains, is recommended for adults, especially for people with high risk of CVD and obesity [48]. In our systematic review, we assumed that obese patients receiving whole grain diet intervention would have lower CVD risk factors, such as body weight, LDL-C concentration, SBP, waist circumference, CRP, insulin resistance index and BMI. However, we only observed a slight reduction in body weight and LDL-C concentration in subjects on whole grains diet, compared with the control group. In addition, subjects on whole grains diet also had a greater reduction in CRP, although the heterogeneity among the studies was relatively large.

In order to investigate the source of heterogeneity, subgroup analysis was conducted for three outcomes using the positive results. For weight and LDL-C data, the subgroup whose participants had another chronic disease besides obesity, such as type 2 diabetes, abnormal plasma cholesterol, showed more significant results. Specially, these comorbidities are in accordance with the diagnosis criteria of metabolic syndrome (MetS). As defined by the United States National Heart, Lung and Blood Institute and by the American Heart Association Consensus Statement, MetS can be diagnosed when a patient has at least 3 of the 4 risk factors, which include abdominal obesity [waist circumference > 102 cm for men, or > 88 cm for women], high triglycerides (≥150 mg/dL), high-density lipoprotein cholesterol (HDL-C) [fasting serum HDL-C <35 mg/dL for men, or <39 mg/dL for women], high blood pressure [BP ≥130/≥85 mmHg], and elevated fasting blood glucose (≥ 100 mg/dL) [49]. MetS was reported as an important contributor for CVD incidence and mortality, as well as for all-cause mortality. Previous studies have demonstrated that whole grains diet can greatly reverse the process of MetS, lower postprandial plasma insulin and cholesterol levels [4, 10, 50, 51]. Our results were in line with these findings, but also showed that whole grains diet can exert more effects on patients with more than one chronic metabolic disorders.

Another factor that was highlighted by the subgroup analysis was well-organized study design. Indeed, studies with positive results in this review showed high quality intervention monitoring. One effective method was “centralized intervention”, which relies on giving educational lessons or standard 7-day cyclical menu before intervention period or during the visits [25, 40]. Certain studies also supervised participants’ diet by providing a 4-day diet record at each visit in a specific nutrition clinic, for which professional dieticians were recruited to assess these diet records and decide whether participants’ diet should be adjusted according to the study design [28, 39]. Precise baseline information collection was also emphasized in these studies, which introduced a run-in period before the intervention period. In the run-in period, participants in both groups were asked to replace their habitual grain products with refined grains only to eliminate the habitual diet influence [28, 39]. In fact, structured run-in period was reported as an important element in clinical trial design, especially in medical studies and clinical trials. Run-in strategy is usually used to diminish the effect of prior treatments, while it has no significant effect on realistic intervention outcomes [52, 53]. The duration of run-in period is still controversial, and this period was designed as 4 to 6 weeks, unequally, in our review.

Discrepancies among studies may also be caused by a variety of whole grains diet interventions. In our review, the diets included barley, oat, wheat, rye and quinoa, and few studies only gave ambiguous definitions. Previous studies showed differences when considering the type of whole grains diet. In a 6-week randomized trial, Suhr et al. reported that whole grain rye, but not wholegrain wheat, significantly lowered body weight and fat mass, compared with refined wheat [54]. In a meta-analysis, Li et al. investigated the effect of buckwheat on CVD risk factors in both human and animals. In human, buckwheat intervention significantly reduced glucose metabolism (0.85 mmol/L, 95% CI[1.31, 0.39]), total cholesterol (0.50 mmol/L, 95% CI [0.80, 0.20]) and triglycerides (0.25 mmol/L, 95% CI [0.49, 0.02]), compared with the control group. However, only triglycerides and total cholesterol showed slight differences in animals, with high heterogeneity [55]. On the other hand, another trial on quinoa suggested that quinoa consumption can regulate glucose response, with only minimal effects on other CVD risk biomarkers [56]. As a result, the discrepancies among studies in this review could be related to different diets, and thus, further subgroup analysis should be conducted based on the type of whole grains diet.

Interestingly, we observed that only few studies investigated plasma alkylresorcinols as a biomarker to quantify the intake of whole grain diet, which can lead to more accurate measurement of the effectiveness of whole grains diet, especially for wheat, rye and barley [27, 33, 57]. Alkylresorcinols are a short-half-life phenolic lipid compounds that are abundant in the outer layer of whole wheat, rye and barley, and is homologues with odd-numbered hydrocarbon side chains [57, 58]. Although its half-life is estimated to be around 5 h, single plasma alkylresorcinols measurement has been shown to be a reliable biomarker for long-term whole grain food consumption [59]. The concentration of alkylresorcinol has been also reported as a sensitive indicator that is correlated with whole grain intake, and it could be used to distinguish between low- and high- whole grain consumers. Besides, it was suggested that there is no difference if the alkylresorcinol concentration is expressed by “nmol/mmol total lipids” or “nmol/L”, which indicates that the concentration of alkylresorcinol is not influenced by lipid distribution [60]. In summary, the concentration of alkylresorcinol should be used as a reliable biomarker for evaluating the true effect of whole grain diet in future studies.

There are some limitations about in this study that are worth to mention. First, only 22 studies including 1865 subjects met our inclusion criteria. Hence, the issue of bias and heterogeneity might not be fully investigated using such as relatively small sample size. Second, some of the included RCTs lacked baseline information and/or outcome data for the comprehensive meta-analysis. Third, the difference in the composition of whole grains in each article may cause deviations in the results. Therefore, the outcomes and overall conclusions should be interpreted with these limitations in mind.

## Conclusion

In conclusion, our study demonstrated that whole grain food can slightly reduce body weight and CRP in obese populations, compared with non-whole grain diet, with not significant effect on other CVD risk factors. This effect was more likely in patients with other chronic metabolic disorders besides obesity. The discrepancies among studies can be explained by the different monitoring approaches and by the types of diets used. To adjust for the effectiveness among diverse types of whole grain diets, plasma alkylresorcinol concentration can be used in future studies as a biomarker to reflect the level of whole grains intake. In addition, further studies should be conducted on more specific subgroups of patients.

## Data Availability

The datasets used and/or analysed during the current study are available from the corresponding author on reasonable request.

## Abbreviations

BMI: Body mass index
BP: Blood pressure
CI: Confidence intervals
CRP: C-reactive protein
CVD: Cardiovascular disease
DBP: Diastolic blood pressure
LDL-C: Low-density lipoprotein cholesterol
MD: Mean difference
MetS: Metabolic syndrome
OR: Odds ratios
PRISMA: Preferred reporting items for systematic reviews and Meta-analyses statement
RCTs: Randomized controlled trials
Revman: Review manager
SBP: Systolic blood pressure
